# Gold Nanowires/Fibrin Nanostructure as Microfluidics Platforms for Enhancing Stem Cell Differentiation: Bio-AFM Study

**DOI:** 10.3390/mi11010050

**Published:** 2019-12-30

**Authors:** Hadi Hashemzadeh, Abdollah Allahverdi, Mohammad Ghorbani, Hossein Soleymani, Ágnes Kocsis, Michael Bernhard Fischer, Peter Ertl, Hossein Naderi-Manesh

**Affiliations:** 1 Department of Nanobiotechnology, Faculty of Biological Sciences, Tarbiat Modares University, Tehran 14115-154, Iran; Hashemzadeh.hadi@gmail.com; 2 Department of Biophysics, Faculty of Biological Sciences, Tarbiat Modares University, Tehran 14115-154, Iran; aallahverdi@gmail.com (A.A.); mohammad.ghorbani@modares.ac.ir (M.G.); Hssnslmni@gmail.com (H.S.); 3 Department of Health Science and Biomedicine, Danube University Krems, 3500 Vienna, Austria; agika.kocsis@gmail.com (Á.K.); michael.fischer@donau-uni.ac.at (M.B.F.); 4 Faculty of Technical Chemistry, Institute of Applied Synthetic Chemistry and Institute of Chemical Technologies and Analytics, Vienna University of Technology, Getreidemarkt 9, 1060 Vienna, Austria

**Keywords:** alternative microfluidics substrate, bio-AFM, stem cell differentiation, gold nanowire, lab-on-a-chip

## Abstract

Organ-on-a-chip technology has gained great interest in recent years given its ability to control the spatio-temporal microenvironments of cells and tissues precisely. While physical parameters of the respective niche such as microchannel network sizes, geometric features, flow rates, and shear forces, as well as oxygen tension and concentration gradients, have been optimized for stem cell cultures, little has been done to improve cell-matrix interactions in microphysiological systems. Specifically, detailed research on the effect of matrix elasticity and extracellular matrix (ECM) nanotopography on stem cell differentiation are still in its infancy, an aspect that is known to alter a stem cell’s fate. Although a wide range of hydrogels such as gelatin, collagen, fibrin, and others are available for stem cell chip cultivations, only a limited number of elasticities are generally employed. Matrix elasticity and the corresponding nanotopography are key factors that guide stem cell differentiation. Given this, we investigated the addition of gold nanowires into hydrogels to create a tunable biointerface that could be readily integrated into any organ-on-a-chip and cell chip system. In the presented work, we investigated the matrix elasticity (Young’s modulus, stiffness, adhesive force, and roughness) and nanotopography of gold nanowire loaded onto fibrin hydrogels using the bio-AFM (atomic force microscopy) method. Additionally, we investigated the capacity of human amniotic mesenchymal stem cells (hAMSCs) to differentiate into osteo- and chondrogenic lineages. Our results demonstrated that nanogold structured-hydrogels promoted differentiation of hAMSCs as shown by a significant increase in Collagen I and II production. Additionally, there was enhanced calcium mineralization activity and proteoglycans formation after a cultivation period of two weeks within microfluidic devices.

## 1. Introduction

Given the increasing demand for stem cells for cytotoxicity screening, disease modeling, pharmaceutical compound testing, and cell-based therapies [1,2,3], scientists will need well-characterized stem cell cultures that exhibit controlled expansion with the ability to direct stem cell differentiation reliably [1,2,3]. Herein, microfluidics and organ-on-a-chip technology and its ability to precisely and reproducibly control the spatial and temporal microenvironment of single stem cells in combination with cost-effective solutions have provided a variety of new and exciting possibilities [1,4,5,6]. A range of biochemical and structural cues, as well as mechanical stress, electromagnetic forces, light and ultrasound stimulation, can influence and govern stem cell fate [2,7,8,9,10]. An important aspect of stem cell research is the availability of well-characterized and validated pluripotent stem cell cultures not only for medical use but also in a variety of in vitro cell-based assays that are routinely used in stem cell research [11]. However, the drawbacks of using conventional cell-based assays include their limited reproducibility, reliability, and robustness that can lead to experimental inconsistencies and difficulties in cell culture propagation and stem cell differentiation [12]. In past years, numerous studies have investigated the effect of engineered microenvironments on stem cell differentiation, as well as proliferation [2,10,13,14]. Several synthetic and natural polymers have been engineered with the specific purpose of controlling and guiding stem cell proliferation, as well as differentiation [10]. Nano-topography and elasticity have emerged as powerful candidates for the guidance and manipulation of cells [4,7,15]. In particular, natural polymers are of interest due to their ability to provide cells with highly complex structures in vitro and their good biocompatibilities [1,16]. One particularly interesting natural polymer is fibrin. Fibrin is characterized by low cytotoxicity, excellent biocompatibility, enhanced cell adhesion, and cell-mediated degradation kinetics [6,17]. However, poor knowledge of the mechanical properties of fibrin, constitute one of the most important limitations of this natural biomaterial [6].

**Figure 1:**
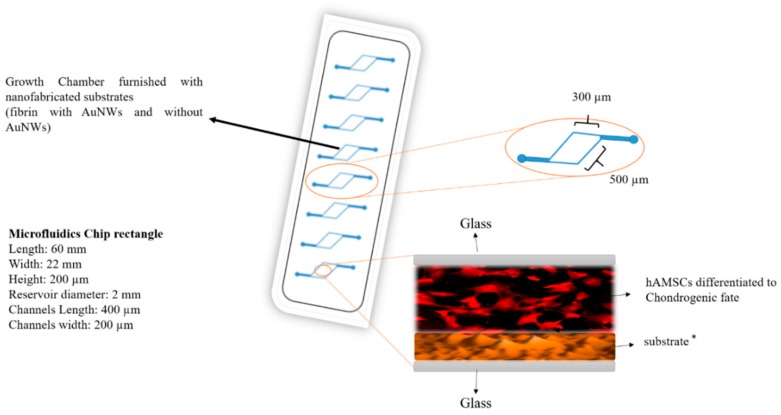
Schematic illustration of microfluidic biochips used in the study containing gold nanowire laden fibrin hydrogels as an advanced biointerface for hAMSC cultivation. * are four different substrates in special channels such as fibrin with AuNWs (low concentration of fibrinogen and thrombin), fibrin without AuNW (low concentration of fibrinogen and thrombin), fibrin with AuNWs (high concentration of fibrinogen and thrombin), fibrin without AuNW (high concentration of fibrinogen and thrombin).

Although the controlled manipulation of biointerfaces by incorporating nanoparticles into biomaterials has already been shown, [10,13,15], there is limited knowledge on the induced biophysical changes of the nano-enhanced matrix that modulates the differentiation capacity of stem cells [2,7,8]. For instance, the cardiogenic differentiation of mesenchymal stem cells was regulated tightly by synergistic cues from both the ECM and the embedded nanoparticles [18]. Overall, human mesenchymal cells are highly susceptible to the physico-chemical cues of their surrounding ECM. This makes the cells good candidates for further study [7,15,16,17]. Therefore, the key to defining a stem cell’s fate involves investigating the effects of biophysical parameters such as stiffness, topography, and the role of nanomaterials incorporation on stem cell differentiation. We studied the modulating effect of matrix elasticity and topography on both osteo- and chondrogenic differentiation of human adipose-derived mesenchymal stem cells (hAMSCs). We did this by coating microfluidic devices with a fibrinogenous matrix embedding gold nanowires (AuNWs). Overall, we assessed the effects of elasticity and topography on the ECM deposition of collagen I, II, and proteoglycans, as well as calcium mineralization of hAMSCs. Furthermore, scanning electron microscopy (SEM) and UV-Vis were used to characterize the morphology of AuNWs and to evaluate nanoparticle synthesis. Finally, atomic force microscopy (Bio-AFM) was used to measure the roughness, Young’s modulus, and adhesion force within the scaffold. We believe that the incorporation of nanomaterials such as gold within natural biopolymers presents an attractive strategy to alter the fate of hAMSCs into both osteogenic and chondrogenic lineages. This is especially the case when combined with the advantages of organ-on-a-chip technology. The schematic illustration of the designated microfluidics chip is presented in Figure 1.

## 2. Materials and Methods

### 2.1. Synthesis of Gold Nanowires (AuNWs)

We synthesized gold nanowires (AuNWs), followed by the use of the anisotropic elongation method. Briefly, both the seed and growth solutions were prepared separately. Preparation of the seed solution was conducted by adding sodium citrate (0.25 mM) and chloroauric acid (HAuCl_4_ 0.25 mM) in 20 mL double distilled water. Then, 600 µL NaBH_4_ (0.1 M) was added to the mixture in vigorous stirring till the color turned into a deep red. At this stage, a spherical gold nanoparticle, approximately 4 nm in diameter, was formed and it was stable at 4 °C for a couple of months. To facilitate NaBH_4_ evaporation, the solution was kept for several hours at room temperature. To prepare the growth solution, cetyltrimethylammonium-bromide (CTAB) (0.1 M) was added to 200 mL deuterium-depleted water (DDW), and then HAuCl_4_ (0.25 mM) and ascorbic acid (0.5 mM) were added to the solution. At this stage, the color of the solution turned from deep yellow to pale yellow (Au (III) to Au (I) reduction), and finally, we added nitric acid (70 mM). To increase the efficiency and reach to high aspect-ratio of AuNWs, we aliquoted the solution into three glass vials as follows: 25 mL in glass vials number 1 and 2 and 250 mL in glass vial number 3. The final solution (growth solution) was aliquoted between glass vial number 1 (9 mL), 2 (18 mL), and 3 (173 mL). We started the growth of the AuNWs by adding 1 mL seed solution to glass vial number 1 under vigorous stirring for 15–30 s. Then, under vigorous stirring, a 1 mL solution of glass vial number 1 was transferred to glass vial number 2 and then stirred for 30 s. Finally, after 30–60 s, and under vigorous stirring, 5 mL of the solution of glass vial number 2 was transferred to glass vial number 3, and kept for 2 h under stirring at 37 °C. The solution collected in the centrifuge tube (50 mL) and was left for 1 week without stirring at 37 °C. A brown pellet formed at the bottom of the centrifuge tube, which contained AuNWs %90 usable, and the supernatant was discarded. The pellet was re-suspended in DDW before using it.

### 2.2. Characterization of the AuNWs via SEM

After synthesis of the AuNWs, their characterization was conducted by UV-Vis, as well as SEM. To characterize the nanoparticle using SEM, a drop of water containing AuNWs was placed on an aluminum substrate and incubated until the water evaporated. It was then sputter-coated with a 2 nm layer of gold to prevent charge build-up using sputtering (sputtering model Yar Nikan Saleh^©^, Tehran, I.R.Iran) and then the Au-NW was characterized using a KYKY-EM3200 digital scanning electron microscope (SEM-Model tech, KYKY Technology Development Ltd., Bejing, China).

### 2.3. Thin-Film Layer Preparation in Microfluidics Channels

The template for the microfluidic chip was designed using AUTOCAD 2016 (Autodesk, San Rafael, CA, USA). Then, polydimethylsiloxane (PDMS) sheets were placed in a CAM-1 GS-24 cutter (Roland DGA Corporation, Irvin, CA, USA) and the desired architecture was made, followed by design using the CAM-1 GS-24 cutter controlled by a computer. The PDMS chip and microscope coverslip were plasma oxidized using air plasma (Harrick Plasma, High Power, 45 s), and a glass slide was immediately attached to the PDMS surface and then baked at 70 °C overnight. As shown in Table 1, before cell seeding into the microfluidic devices, fibrin hydrogel (TISSEEL^®^, Baxter, Deerfield, IL, USA), with and without AuNWs, at the defined concentration shown in Table 1, was loaded into the microfluidic channels and then polymerized for 1 h. We note that, due to fibrin behavior, we gently mixed the AuNWs and fibrinogen. Then, before loading into the device, we added the thrombin and gently mixed it with AuNWs and fibrinogen components, and finally, we quickly loaded the mixture into the microfluidic device.

### 2.4. Cell-Based Assays

**Figure 2:**
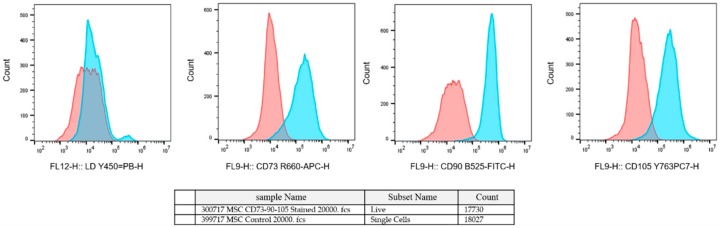
Fluorescence-activated cell sorting (FACS) analysis of hAMSCs with CD90/105/73.

Human amniotic-derived mesenchymal stromal cells (hAMSCs) were isolated from the amniotic sack and cultured at the Danube University Krems. The study was approved by the Ethics Commission of the Medical University Vienna (EK791/2008, EK1192/2015), the University Hospital of Lower Austria (GS1-EK-4/312-2015), and the Danube University Krems (Nr.821/2009, 3 September 2015). The placenta was obtained from a healthy delivering woman per the Austrian Hospital Act (KAG 1982) after written informed consent was obtained. The amniotic membrane was isolated from the placenta and cut into 3 × 3 cm squares, and then the pieces were washed in physiological NaCl solution and digested with dispase (2.5 CU/mL, Becton Dickinson, Franklin Lakes, NJ, USA) for 9 min at 37 °C (Soncini et al. 2007). The slice was incubated in a mixture of collagenase A (1 mg/mL) and DNase I (0.01 mg/mL both from Roche, Basel, Switzerland) for 2 h at 37 °C. To separate the stem cells from the tissue, we centrifuged the mixture at 180 g for 5 min, and the supernatant was strained with a 100 µm cell-strainer. The amniotic cell suspension was centrifuged at 360 g for 10 min, and then they were proliferating in an adherent 2D cell culture in plastic cell culture flasks. The cell culture was maintained within an MSCGMTM (Lonza Group Ltd., Basel, Switzerland) supplemented with 100 U/mL penicillin, 100 µg/mL streptomycin, and 250 ng/mL amphotericin B (all from Gibco, Thermo Fisher Scientific, Waltham, MA, USA) at 37 °C in a 5% CO_2_ humidified environment (Stericycle, Thermo Fisher Scientific). The medium was changed every 3–4 days and the hAMSCs were passaged at 80% of confluence. After the first passage, the amniotic cells were characterized with CD90/105/73 using flow cytometry and positive cells were considered as hAMSCs (Figure 2).

After that, the hAMSCs were detached by Accutane and Trypsin enzymes and then used for seeding into microfluidics devices. Before cell seeding into microfluidics devices, the chip was sterilized under UV irradiation for 1 h, then 5 × 103 cells per mL were seeded into the microfluidics chip. After the expansion of the hAMSCs, the medium was replaced by an osteogenic stem cell differentiation medium (StemMACS OsteoDiff Media, order No. 130-091-678, Miltenyi Biotec GmbH, Bergisch Gladbach, Germany) supplemented by fetal calf serum (FCS) 10% and 1% penicillin/streptomycin. For chondrocyte differentiation, we used the chondrogenic commercial medium (Catalog No: BN-0012.17, bonbiotech company, Tehran, I.R.Iran). During stem cell differentiation on-a-chip, the media were replaced by OsteoDiff and chondrocyte differentiation media every 1–2 days since the chip was kept under CO_2_ (5%), at 37 °C in an incubator. After two weeks of hAMSCs treated using osteo/chondrocyte differentiation media, the samples were used to characterize the differentiation rate.

### 2.5. Nanotopographical Characterization of Fibrin/AuNWs Thin-Film by AFM 

The surface roughness, stiffness, Young’s modulus, and adhesion force of the scaffold in fibrin, with and without AuNWs, was determined by atomic force microscopy (AFM, CoreAFM, NanoSurf Co. Ltd., Lausanne, Switzerland) using the tapping mode probe. The probe comprised a silicon tip with a radius of <10 nm and a spring constant range of 48 N/m. Then, 2 μm × 2 μm topographical images were scanned at 0.8 Hz, at a set point of 0.7 V and a resolution of 256 pixels. At least 3 points of contact were analyzed for each sample substrate. To determine the different roughness in surfaces, we applied different thin-film layers in an area of 100 µm^2^ to scan, and then the images of the surface in a non-contact mode were taken. To describe the roughness of the surfaces, the topography of the surface and the roughness parameter of the surface, Sa; which is the area average or the distance between the highest and lowest point of the surface irregularities, were shown and calculated by built-in software (NanoSurf™ CoreAFM Software, version 3.8.1.4, Lausanne, Switzerland).

### 2.6. Immunocytochemical Staining and Measurement of the Proteoglycans Formation and Calcium Deposition 

Immunocytochemical staining of the differentiated hAMSCs on the different substrate was performed using primary antibodies against collagen type I and II (Abcam; mouse monoclonal, ab6308, 1/1000 dilution, and Abcam; Rabbit polyclonal to Collagen II, ab34712 1/200 dilution). The samples were carefully and gently rinsed with phosphate-buffered saline (PBS) several times, and then fixation and permeabilization were carried out using a solution of 4% paraformaldehyde and 0.5% Triton X100. To block the nonspecific antibodies, we applied 3% bovine serum albumin (BSA) then washed it with PBS 1x. Staining was done using Alexa-flour secondary antibody (1/200 dilution monoclonal goat anti-rabbit IgG) for osteogenic and Alexa Fluor® 594-conjugated goat anti-rabbit polyclonal IgG (ab150080, abcam, Cambridge, UK) was used for chondrogenic. It was then washed with PBS, and stained by DAPI (4′,6-diamidino-2-phenylindole). Finally, the stained differentiated hAMSCs cells were observed using an Olympus IX83, and the images were analyzed using Fiji software for optical density measurement. The evaluation of osteogenesis and chondrogenesis on fibrin, with or without AuNWs, was carried out using alizarin red (2%) and toluidine blue, respectively. The medium was removed from each cell chamber, then washed several times with PBS and then fixed with 4% paraformaldehyde (the paraformaldehyde was heated before using in a 37 °C water bath). The microfluidics devices containing the differentiated hAMSCs were immersed in the alizarin red and toluidine blue solutions. Afterward, to remove the nonspecific staining, the samples were washed several times gently with DDW. Finally, the images of the samples were captured by an Olympus IX71 microscope (Olympus Ltd, Tokyo, Japan), and the was data analyzed by the Fiji software (version 1.52i, Fiji, Madison, WI, USA) using the optical density measurement.

### 2.7. Bio-AFM Study of Scaffold Stiffness, Young’s Modulus, and Adhesion Force

To evaluate the physical and mechanical properties of nanostructured architectures, after gelation of the substrate, the specimens were carefully rinsed several times by DDW to remove the existing salt. Then, the specimens were immersed into DDW and Bio-AFM mode (AFM, CoreAFM, NanoSurf Co. Ltd, Lausanne, Switzerland) was used to measure the stiffness, Young’s modulus, and adhesion force of scaffolds. Bio-AFM analyses were obtained with a spectroscopy wizard using the Stat0.2LAuD cantilever (NanoSurf Co. Ltd, Lausanne, Switzerland). Spring constant and sensitivity were fixed to 0.02 N/m and 0.075 μm/V, respectively. To measure the surface mechanical properties, force curves were obtained at the different regions of the surface. To make the obtained data comparable, we obtained all force curves at the same loading rate and the ramp size of the force curves was 5 μm. To map the stiffness distributions of local areas on the surface, we obtained the arrays of force curves (8 × 8). The original force curves were exported to the AtomicJ software (version 1.7.3, NanoSurf Co. Ltd, Lausanne, Switzerland) [19]. The sphere (Hertz) model was used to calculate the cellular Young’s modulus from the force curves, as described in Reference [20].

### 2.8. Statistical Analysis

The images data collected from the microscopy technique were subjected to analysis of variance. The means were compared using Duncan’s range test at *p* = 0.05. The means ± SE were used to compare the data. Mean optical density and fluorescence intensity of pixel by pixel of the visualized differentiated hAMSCs were analyzed using Fiji (version 1.52i, Fiji, Madison, WI, USA) and GraphPad Prism software (version 8.0.2, Prism Corporation Pvt Ltd, San Diego, CA, USA). 

## 3. Results

We applied lab-on-a-chip technology to investigate the role of microenvironments that significantly influence stem cell fate. The lab-on-a-chip approach can design complex physiological environments that are suitable for stem cells by using nano-patterned hydrogels. These hydrogels physiologically mimic a 3D extracellular matrix, the modification of physicochemical properties of the surface, and accurately control the flow [1,6,13,21]. In addition, microfluidics shows our knowledge about the role of nanostructured pattern scaffolds for stem cell analysis in a well-controlled situation [1]. An important issue associated with employing nanomaterials to advance biomaterials is related to enhancing the quality of the biomaterials [2,9,13,18,21,22,23]. In other words, there is a need for an advanced substrate in the microfluidic channels used for cell-on-a-chip research. Given the drawbacks of PDMS as a microfluidics cell substrate, an alternative is needed. 

### 3.1. Surface Characterization of AuNWs and Substrate

**Figure 3:**
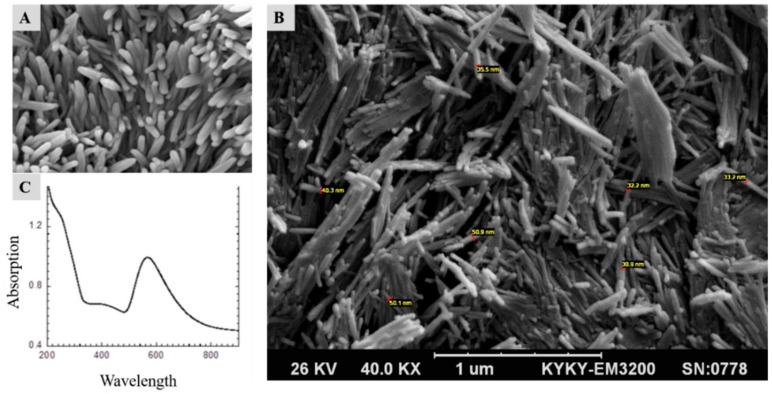
Scanning electron microscopy (SEM) and ultraviolet-visible (UV-Vis) spectrum analysis of the AuNWs. (**A**) and (**B**) scanning electron microscopy and (**C**) UV-Vis spectrum of the synthesized AuNWs. The length of the nanowires is around 1 µm and the diameter size is less than 50 nm.

**Figure 4:**
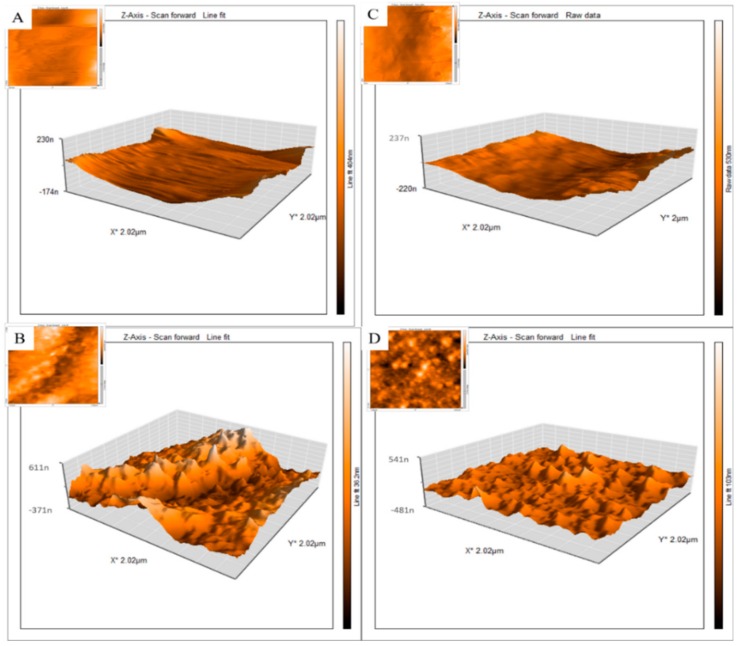
2D and 3D atomic force microscopy (AFM) images of lab-on-a-chip fibrin scaffolds with and without AuNWs: (**A**) S_1_/Au^−^; (**B**) S_1_/Au^+^; (**C**) S_2_/Au^−^ and (**D**) S_2_/Au^+^. As shown in Table 1 (S_1_ = stiffness 1, S_2_ = stiffness 2). Au^+^ = with AuNWs, Au^−^ is without AuNWs as indicated in Table 1. n = 6 (at least) and p = 0.05. Significant differences were observed between (**A**,**B**) samples, as well as between (**C**,**D**).

The employed gold nanowires were synthesized in-house, where they gave novel properties to the fibrin proteins. As shown in Figure 3 and Figure 4, the AuNWs and scaffolds were characterized using a scanning electron microscope (SEM), Bio-AFM, and Ultraviolet-visible spectroscopy (UV). Furthermore, we designed and fabricated a microfluidics device with a nanostructured substrate that included AuNWs and fibrin proteins. We used it to study the effect of different concentrations of AuNWs at various ratios of fibrin components (fibrinogen and thrombin) on hAMSCs differentiation. The results showed that the UV-Vis spectrum of synthesized AuNW samples (Figure 3A) had a distinct absorption wavelength at 540 nm, which belonged to the transverse plasmon resonance mode. While the micrometers length of the AuNWs causes the longitudinal mode shift beyond the visible area, the region of 600–700 nm showed a shoulder, which could be related to other byproducts. Additional SEM images shown in Figure 3A,B reveal the nanometer size of the rod-like shape of the gold nanowires. The SEM images revealed an average of approximately 30–50 nm. 

Table 1 shows that in a next step, different ratios of fibrin protein (i.e., different concentrations of fibrinogen/thrombin) with or without AuNWs are constructed and loaded into microfluidic channels. Finally, the hAMSCs were seeded into the microfluidics device to study the impact of different substrates on the control of the hAMSCs fate. Figure 4 shows 2D (insets) and 3D AFM images of fibrin, with and without AuNWs, at different concentrations of fibrinogen/thrombin surfaces, respectively. Results of the Bio-AFM study demonstrated a 3–5-fold increase in surface roughness in the presence of the AuNWs. 

**Figure 5:**
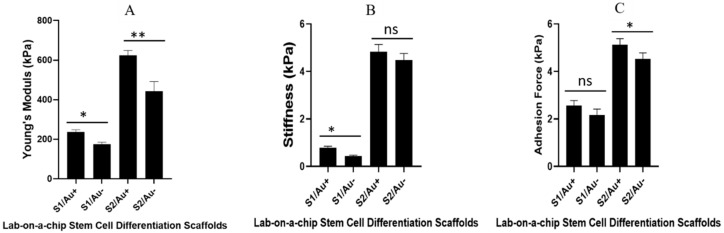
Bio-AFM measuring of (**A**) Young’s modulus; (**B**) stiffness; and (**C**) adhesive force of the microfluidics channels containing fibrin, with and without AuNWs scaffolds. The * is significance at p = 0.05, ** is significance at p = 0.01, and ns is non-significance. Significant differences were observed among all mechanical properties (soft and stiff substrate with and without AuNWs). n = 3 and p = 0.05.

As cells are living organisms in a bio-environment, it is helpful to compute stiffness, adhesion force, and Young’s modulus using Bio-AFM. Understanding these parameters of substrates in bio mode yields more information on cell-substrate interactions. Figure 5 shows the result of Bio-AFM measured stiffness, Young’s modulus, and adhesive force. Our results showed that Young’s modulus of the high ratio of fibrin components (thrombin and fibrinogen) were higher than the substrates with a low ratio of fibrin components. However, we observed a different result for the substrate with AuNWs, as compared to the substrate without AuNWs. We also show other mechanical properties measured by Bio-AFM such as stiffness and the adhesive force of substrates.

Several studies have reported the effect of Young’s modulus, stiffness, roughness, and adhesive strength of the substrate on stem cell differentiation [7,24]. The stem cells on a stiffer substrate express the osteogenic marker, while a softer scaffold promotes the chondrogenic marker in stem cells [7]. Based on our result, the microfluidic channels containing a stiffer substrate (especially with AuNWs) with high Young’s modulus, stiffness, and adhesion force promoted osteogenic differentiation. This was observed for chondrocyte differentiation in the soft substrate. 

### 3.2. Effects of Mechanical Properties on hAMSCs Differentiation 

**Figure 6:**
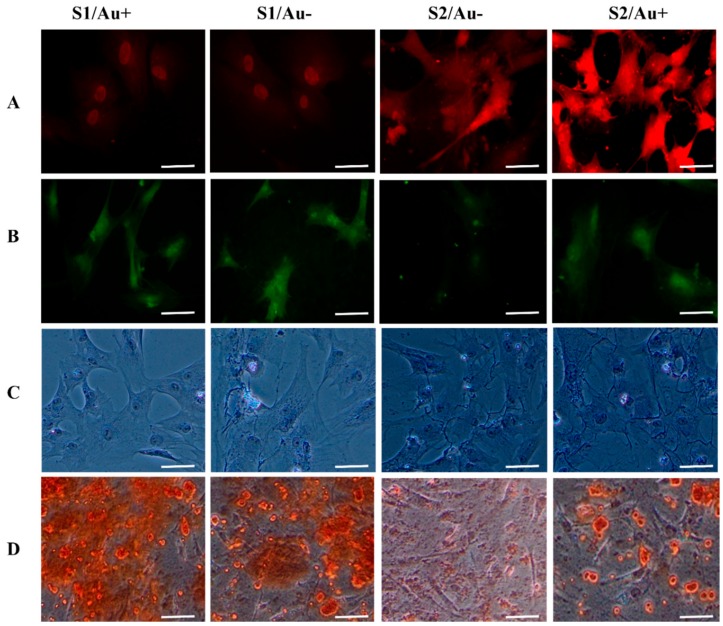
Immunohistochemistry analysis, calcium deposition, and proteoglycan formations of the osteogenic and chondrogenic specific markers in hAMSCs seeded into microfluidics channels containing fibrin scaffolds with and without AuNWs. The differentiated hAMSCs stained by collagen II and toluidine blue characterize the chondrogenic lineage, and collagen I antibody and alizarin red characterize the osteogenic lineage. Differentiated hAMSCs seeded on different substrates (see Table 1): (**A**) differentiated hAMSCs stained with collagen II antibody, (**B**) differentiated hAMSCs stained with collagen I antibody, (**C**) the proteoglycans formation stained with toluidine blue and the differentiated hAMSCs, and (**D**) differentiated hAMSCs seeded on microfluidics substrates stained with alizarin red. Scale bar for rows number 1–3 = 30 µm and for row number 4 = 50 µm.

**Figure 7:**
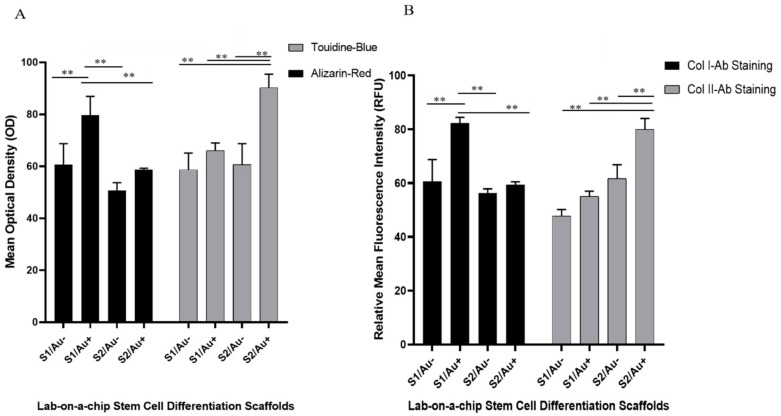
Effect of substrate composition (stiffness and presence of AuNW) on the differentiation of hAMSCs to osteo/chondrogenic fate characterized by (**A**) alizarin red and toluidine blue staining and (**B**) immunohistochemical staining. hAMSCs were differentiated on microfluidic channels containing fibrin scaffolds of varying stiffness with and without AuNW. The relative mean fluorescence intensity (RFU) was applied to characterize the expression of cell surface markers (antibody staining) of osteo/chondrogenic. The mean optical density was used to characterize the calcification and proteoglycans formation. S1/Au^+^ is a low concentration of fibrin components with AuNWs, and S1/Au^−^ is a low concentration of fibrin components without AuNWs. S2/Au^+^ is a high concentration of fibrin components with AuNWs, and S2/Au^−^ is a low concentration of fibrin components without AuNWs. ** Significant difference between the two groups (*p* < 0.01).

Differentiated hAMSCs on chondro/osteogenic fate should express cell surface markers such as collagen I and II, as well as the mineralization of calcium and proteoglycans formation [7,21,25,26]. We evaluated the frequency of cell surface markers expression, calcium deposition, and proteoglycan formation two weeks after the in vitro cell seeding of hAMSCs into the microfluidics devices. In our study, we used collagen I cell surface marker and calcium deposition for osteogenic differentiation and collagen II cell surface marker and proteoglycans formation were used for chondrogenic differentiation. Collagen I and II antibodies are known as one of the most important cell surface markers in stem cell differentiation of osteogenic and chondrogenic fate, respectively. We found that collagen I and II antibodies activation in differentiated hAMSCs was induced by fibrin containing the AuNWs substrate to a significantly greater degree than by fibrin without the AuNWs substrate. The levels of cell-surface markers, calcium mineralization, and proteoglycans formation in the hAMSCs seeded on the microfluidics channels with the fibrin substrate with AuNWs were significantly higher than the fibrin substrate without AuNWs (Figure 6). It appeared that incorporating the AuNWs into the fibrin proteins led to the enhancement of the hAMSCs guiding to a specific lineage. Mixing the AuNWs with fibrin at different stiffness could significantly enhance the hAMSCs differentiation into the desired lineage (Figure 7). 

Our results indicated that the chondrocyte differentiation medium improved on fibrin/AuNWs surfaces with a low concentration of fibrin components (soft substrate). Moreover, hAMSCs differentiation of osteogenic fate improved with the fibrin/AuNWs containing high concentrations of fibrin components (stiff substrate). The microfluidics channels containing scaffolds with the lowest ratio of fibrin components (5 µg/5 U per mL of fibrinogen/thrombin) facilitated a significant expression of chondrocyte cell surface markers. Meanwhile, between both soft scaffolds (with and without AuNWs) of fibrin, the fluorescence intensity of the chondrocyte cell surface marker was greater than the scaffolds without AuNWs (Figure 5 and Figure 6). 

Furthermore, in the case of osteogenic cell surface marker level, we observed that the level of collagen type I was higher in hAMSCs seeded on the scaffolds with a high ratio of fibrin components (50 µg fibrinogen /50 U thrombin per mL) than the scaffolds with the lowest ratio of fibrin components. Meanwhile, at this ratio of fibrin components, the expression of the cell surface marker indicated that hAMSCs are guided towards an osteogenic lineage, and in scaffolds with AuNWs, these have a significant difference than scaffolds without AuNWs. These findings were confirmed by alizarin red and proteoglycans formation staining analyses (Figure 6 and Figure 7). The lab-on-a-chip channels containing cellular constructs with the highest fibrin ratio showed matrix mineralization (calcium deposition) with intense alizarin red staining. This was in contrast to microfluidics channels with the lowest fibrin ratio cultures, which observed low calcium deposition stained with alizarin red. In addition, similar to cell surface markers, the constructs with AuNWs improved the mineralization of calcium in comparison to the constructs without AuNWs. The same results of collagen type II expression were observed for proteoglycan formation, where the scaffolds without AuNWs had a low optical density in terms of proteoglycan formation. In other words, the substrate with AuNWs could improve chondrocyte markers formation. This result was not observed in both scaffolds with AuNWs (i.e., the lowest and highest fibrin components ratio). This meant the high rate of proteoglycan formation was observed in the fibrin/AuNWs scaffold with a low ratio of fibrinogen and thrombin. Moreover, many studies have demonstrated the morphological changes during the osteoblast and chondrocyte differentiation process [25,26]. In our study, a round polygonal appearance with small nodules for osteogenic lineage, as well as globular and round cells for chondrocyte fate, started to appear after 2 weeks. 

## 4. Discussion

Chemical cues are vital for guiding stem cell lineage specification. For instance, scaffold properties such as elasticity and topography are known to modulate cellular behavior. Nonetheless, the characterization of specific physical cues, which are important in driving cellular behavior, remains a challenge [3,7,12,27]. Fibrin is a natural polymer that has microstructural properties facilitating cell survival, growth, and differentiation and it is extensively used in tissue engineering and regenerative medicine applications [16,17]. In this study, the effect of matrix elasticity and nanoparticle incorporation on hAMSCs was analyzed based on cell surface marker level, calcium mineralization, and proteoglycans formation to identify the effect of fibrin properties on stem cell differentiation. Since fibrin elasticity can be tuned by varying the two components, it provides an optimal tool for assessing the influence of matrix elasticity on stem cell lineage specification [16,17,27,28]. Interestingly, soft fibrin scaffolds induced chondrogenic differentiation while stiffer scaffolds led to a dramatic increase in cell surface marker levels of collagen type I and calcium deposition, indicating a commitment to the osteogenic lineage. Subsequently, the AuNWs were incorporated into the fibrin scaffolds to enhance hAMSCs differentiation. In this setup, we observed a strong correlation between cell substrate stiffness and nano/microstructural features and with differentiation patterning of the hAMSCs [27,29,30], resulting in accelerated the hAMSCs differentiation. Again, soft substrates were associated with the chondrogenic differentiation of hAMSCs, while stiff substrates improved osteogenic differentiation, further substantiating the claim that incorporation of the AuNWs into fibrin scaffolds led to better guidance of stem cell fate [31]. Mechano-sensing of scaffolds by stem cells is highlighted by signaling of the integrin-mediated focal adhesion [31]. In the case of cell-ECM attachment of tissue or cell culture scaffolds, activation of the integrin receptors leads to activation of intracellular tyrosine kinase, as well as phosphatase signaling. Activation of downstream biochemical signals is important for the regulation of gene expression and stem cell fate [7]. Here, we have found that the hAMSCs have different fates on various lab-on-a-chip architecture substrates. The relationship between AuNWs concentration and fibrin stiffness provides a concept for improving artificial nanostructured substrates to induce hAMSCs differentiation via interactions between the hAMSCs and soft or stiff micro/nanostructured substrates to ultimately guide stem cell fate [3,31]. Interaction of gold nanoparticles with the MSCs membrane is known to induce mechanical stress and as a result, p38 MAPK signaling activation and overexpression of osteogenic specific genes [10]. The nano/microenvironment is known to affect cellular behavior, induce morphological changes, and alter gene expression. Activation of the integrin-mediated intracellular signaling pathway by clustering of integrin, which regulates stem cell differentiation, is one of the well-known roles of the nano/microenvironment niche [15,32,33]. Our result suggests that AuNWs could mechano-transduction affect hAMSCs. Thus, certain cellular signaling pathways occur from nano-topographical cues and regulate the hAMSCs fate. In addition, the synergistic effect of soft and stiff fibrin substrates, with and without AuNWs, highlights the influence of substrate stiffness on hAMSCs differentiation, where osteogenesis needs a stiff substrate while chondrogenesis needs a soft substrate. 

## 5. Conclusions

Although PDMS has numerous benefits in microfluidics applications, it has drawbacks in microfluidics cell-based assays, and therefore, needs functionalization. Hence, the lab-on-a-chip cell-based assay needs a biocompatible substrate to enhance cell adhesion, proliferation, and differentiation. In the present study, we investigated whether the mechanical properties of the substrate, mainly nanotopology, had a pivotal role in hAMSCs differentiation. Microfluidics substrates were fabricated by mixing the AuNWs and fibrin components and then injected into microchannels. Past studies have demonstrated PDMS functionalization by a chemical cross-linker and protein attachment on the PDMS surface for microfluidics cell-based assays. In this study, the furnished microchannels by the nanofabricated substrate showed that they could be potentially used as novel microfluidics stem cell-based assays. Moreover, we demonstrated that the addition of gold nanowires to the fibrin matrix reliably guided hAMSCs differentiation in biochips by altering the physical properties of the respective biointerface. Our study showed that AuNWs promoted the osteogenic differentiation on stiff microfluidics substrate, while the AuNWs enhanced chondrogenic differentiation of hAMSCs on the soft microfluidics substrate. The ability to readily tune the stiffness and nanostructure of any hydrogel matrix within organ-on-a-chip systems offers unprecedented flexibility to manipulate the biological niche. For instance, the combinatorial effects of matrix stiffness and shear force conditions on stem cells can now be investigated in parallel using similar culture conditions. In our study, we used a tunable AuNWs-fibrin substrate to introduce a new platform for stem cell studies. These changes in the AuNWs-fibrin surface profile contributed to the hAMSCs adhesion and differentiation. This study has shown that altering mechanical properties (stiffness, Young’s modulus, adhesive strength, and nanotopology of the substrate) can profoundly change the hAMSCs differentiation profile. The novel and simple methodology could help to enhance stem cell differentiation, as well as microfluidic cell-based assays for long-term stem cell analysis.

## Figures and Tables

**Table 1:** The ratio of fibrin hydrogel components and concentrations of AuNW mixing with fibrin hydrogel. + and − represents the microfluidics channels containing scaffolds with and without gold nanowire, respectively. S1 and S2 represent different stiffness.

Substrate Name	Component Name	Fibrinogen (mg/mL)	Thrombin (U/mL)	AuNW (mg/mL)
Stiffness 1	S1/Au^+^	50	50	3
	S1/Au^−^	50	50	0
Stiffness 2	S1/Au^+^	5	5	3
	S1/Au^−^	5	5	0
